# The impact of distance education on nursing students course performance in a sino-foreign cooperative program during the onset of COVID-19: a quasi-experimental study

**DOI:** 10.1186/s12912-022-01136-1

**Published:** 2023-01-13

**Authors:** Yu Zhang, Ning Zhang, Hongyuan Liu, Yinshi Kan, Yan Zou

**Affiliations:** School of Nursing, Yangzhou University, Jiang Yang Road 136, Yangzhou, Jiangsu Province China

**Keywords:** Distance education, COVID-19, Fundamentals of nursing, Academic self-efficacy, Academic engagement

## Abstract

**Background:**

The outbreak of COVID-19 changed many studies’ teaching mode in higher education profoundly, including nursing. This study evaluated the impact of distance education on the course performance of nursing students in a nursing fundamentals course during the epidemic of COVID-19.

**Methods:**

This is a comparative prospective and retrospective quasi-experimental study. Nursing students in a Sino-foreign cooperative program were allocated to either an intervention group (distance education, *n* = 48) or control group (face-to-face teaching, *n* = 36). A self-efficacy questionnaire, an academic engagement scale and grades of the final written examination were used to evaluate the students’ self-efficacy, academic engagement and academic performance, respectively. The data in this study were analyzed by two independent sample t-tests and the Chi-square test.

Students experiencing distance teaching had worse academic performance (*p* = 0.001) and lower levels of learning behavior self-efficacy (*p*<0.05). The total score of academic engagement (*p* = 0.04) for students experiencing distance teaching were significantly lower than the scores of those students in the control group.

**Conclusions:**

In the context of COVID-19, nursing students conducted using distance education had poor course performance.

**Supplementary Information:**

The online version contains supplementary material available at 10.1186/s12912-022-01136-1.

## Background

The outbreak of COVID-19 has profoundly changed our lives to varying degrees. The social constraints and distances caused by the pandemic have shaped our perceptions of social relationships and behaviors in different ways. Like many aspects of daily life, education has undergone fundamental changes. To ensure the health and safety of teachers and students, the China Ministry of Education decided to postpone the opening of the spring semester in 2020 and required universities to use online courses and resources to support online teaching activities. Thus, our school of nursing has adopted distance learning to ensure instructional progress and quality during the epidemic prevention and control period.

Distance education can be divided into synchronous education and asynchronous education. Synchronization technology allows “real-time” interaction between teachers and students (e.g., audio conferencing, video conferencing, Internet chat). Asynchronous technology involves time delay from instruction to its reception (e.g., email, early video recording, forums) [1]. The implementation of distance education in nursing teaching is conducive to the integration of quality teaching resources and brings convenience to learners. At the same time, distance education enriches teaching methods, and teachers can make use of online technologies and platforms to present teaching content in a more vivid way [2]. In this special period, distance education has broken through the confinement of teachers and students caused by the virus to provide the dissemination of knowledge. However, it may also have drawbacks, such as reduced teacher-student communication, delayed feedback, and the inability to effectively supervise students [3]. In addition, multi-person distance online education platforms have high network requirements, especially for live online teaching where there are frequent network failures, and these situations both affect the teaching schedule and reduce the online teaching experience [4]. Nursing, as a highly practical subject, requires experimental classes. Distance education cannot meet the requirements for apprenticeships and internships, resulting in a lack of clinical nursing practice teaching content [5].

In the literature, we can see that distance education has been applied in several places. A qualitative study of 60 medical students from Saudi Arabia showed that online learning is popular and that students recognize some of the advantages of this method of education, such as better time utilization [6]. Siah et al. [7] reported students undergoing online-tutorials attained a higher level of knowledge. However, students said they also faced a variety of challenges, including methodological issues, content perception issues, technical and behavioral issues, and online exams [6]. Another cross-sectional survey was conducted among medical students from 13 medical schools in Libya. A total of 54% of the respondents thought that interactive discussion could be realized through online learning. However, only 21% agreed that e-learning could be used in clinical practice, while 55% disagreed, and 24% were neutral [8]. Therefore, it is very important to evaluate the availability of distance learning methods and determine their feasibility and adequacy for medical students.

Academic engagement is an active and fulfilling mental state associated with learning [9], characterized by vitality, focus, and dedication, and mainly includes behavioral, cognitive, and emotional engagement. In addition to increasing student satisfaction and maintaining good physical and mental states, academic engagement is also an important predictor of student academic achievement [10], enhancing professional maturity and strengthening the quality of education. The meta-analysis by Chang et al. showed that academic self-efficacy significantly and positively predicts academic engagement and is one of the important factors of academic engagement [11]. Students with high academic self-efficacy are confident in their abilities, easily identify their own strengths and potential, and believe they can successfully master the learning content to achieve expected learning outcomes. Based on this, we decided to use academic engagement, academic self-efficacy, and academic performance to provide feedback on distance learning.

It is worth noting that, in order to cultivate international nursing talents, a group of students from our school of nursing has enrolled in the Chinese-foreign cooperative education program. Chinese-foreign cooperative education is quite different from traditional education in terms of teaching mode, teaching content, training objectives, and management methods [12]. All the courses are taught in English through classes.

Therefore, this study aimed to examine the impact of distance education on course performance, academic engagement and academic self-efficacy, of students in the nursing fundamentals course during the epidemic of COVID-19. The following research questions are: (1) What are the knowledge mastery situation of students who have received education via online? (2) How do students perceive the academic engagement and self-efficacy via online learning?

## Methods

### Setting and sample

A comparative prospective and retrospective quasi-experimental design was carried out for this study. The sample was composed of students in the Sino-foreign cooperative program between the School of Nursing of Yangzhou University and the School of Health Sciences, University of Hull. In the prospective group, referred to as the ‘distance learning format’ group, participants were first-year students enrolled in 2019 (*n* = 48) who were taking the Fundamentals of Nursing I course. A retrospective control group, referred to as the ‘control group,’ were first-year students enrolled in 2018 (*n* = 36) who were taking the Fundamentals of Nursing I course. Students in the Sino-foreign cooperative program are taught in English in all courses. Admission scores for the nursing program in our university are similar from year to year, so students in these two groups began with a similar knowledge base. The 36 students enrolled in 2018 learned the course material through campus-based formats (face-to-face formats) and the 48 students enrolled in 2019 learned the course material through a distance-learning format. Regardless of the setting, students were taught the same content and fundamentals by nursing faculty members with identical assessment objectives.

### Ethics approval

This study was approved by the Yangzhou University Nursing School Ethics Committee. Students who were included in the study offered written informed consent.

### Description of the Fundamentals of Nursing 1 course

The content of the Fundamentals of Nursing I course includes infection control, sleep and pain management, client hygiene, vital signs assessment, circulatory and ventilatory support, nutrition, mobility, and safety. The course was team-taught by seven faculty members. Since this course is the only specialized basic course in the spring semester, the academic engagement and self-efficacy of students are usually evaluated in this semester to promote the improvement of subsequent teaching methods.

### Fundamentals of Nursing 1 course offered in face-to-face modality

The face-to-face format was held in the classroom and was mainly didactic with patient cases incorporated throughout the lecture to engage students in discussion and enhance the learning of the material. Didactic materials and learning objects were given to students before the lectures. Didactic materials covered news videos, industry technical standards, scientific articles, reference books, presentations of lectures of teachers and so on. In class, teachers usually presented the objectives, the key points and difficulties of the lessons first and then delivered this content as a lecture. News videos related to the lessons and some real clinical cases were usually used to introduce the content of this lesson. After learning the lecture, students then discussed topics with each other under the guidance of the teacher during class. Discussion topics included how to formulate nursing measures according to the case, putting forward their own views on some clinical phenomena and so on. After class, the teacher assigned corresponding homework for students to complete independently. Homework is usually included reading industry technical standards, scientific articles, and reference books, finishing some exercises, handing in some simulated operation videos and so on. Before the next class, the teacher would comment on the homework of the previous class. The Supplementary Table 1 presents classes held in the course and how each activity was offered in face-to-face modality. In this modality, students attended two sessions at a time, each of 45 min, for a total of 28 times.

### Fundamentals of Nursing 1 course offered in a distance education modality

The distance education modality was asynchronous and took place on the Yangzhou University Network Teaching Platform, where didactic materials and homework were posted and where class activities could be completed. The platform also offered a section where students could ask questions about knowledge points from class or topic discussion.

The weekly course content for distance learning was the same as that of the face-to-face format, but the delivery of the format was different. Before each class, teachers needed to prepare all kinds of teaching materials posted on the platform. First, a lecture guide list was posted to the platform as an MS Word document, which informed students of the knowledge points to be learned in the class, the corresponding learning materials, the approximate amount of time it takes to learn each knowledge point, and the learning objectives; each knowledge point was arranged according to the learning order. Secondly, some videos of the key points and difficulties of the lesson were provided by the teacher, with each video tending to be 15 to 20 min in length. These were also uploaded to the platform. Other teaching materials related to the course content were also posted to the platform, including news videos, industry technical standards, scientific articles, reference books, and presentations of lectures by the teachers. Finally, teachers posted homework assignments similar to homework given to students in the face-to-face format. All the above materials were uploaded to the platform 48 h before the lesson began. The supplementary table 1 presents lessons held in the course and how each activity was offered in distance education modality.

During the asynchronous education, firstly, the teacher in charge of the lesson uploaded a lecture guide list that can help students know how to use the materials posted on the platform and understand the difficulties and focus of learning. When students have studied all the materials including videos of the key points and difficulties of the lesson, and the teaching materials related to the course content, like news videos, real clinical cases, presentations of lectures of teachers and so on, they need to complete the homework which is same to those in face-to-face modality. If the student had a task to discuss in class or had a question about the content of the study, it can be raised in the question-and-answer section of the online platform. After students accomplished the learning tasks on the platform independently, teachers answered the questions or discussed the topics related to the lesson with students in the question-and-answer section of the online platform within the time set by the teacher.

### Data collection and study outcomes

Academic performance was assessed by grades of the final written examination. For the examination, the difficulty of the written examination and teachers who marked test papers in face-to-face format and distance education format was similar. The grades ranged from 0 to 100, with ≥ 40 being a passing mark.

Academic self-efficacy was assessed by a self-efficacy questionnaire before the start of the course and after the final lesson. The self-efficacy questionnaire was developed by Pintrich et al. [13] and translated and revised into Chinese [14]. The questionnaire was divided into 22 items in 2 self-efficacy dimensions, which included learning ability and learning behavior. Each dimension comprised 11 items that were measured by a 5-point Likert scale, from 5 to 1, indicating that the respondents strongly agreed, agreed, generally agreed, disagreed, or strongly disagreed with the question. The total score ranged from 22 to 110. The higher the total score, the higher the sense of academic self-efficacy. The total Cronbach's α coefficient of the scale was 0.815, the retest reliability was 0.87, and the internal consistency of each dimension was 0.85 and 0.71. Self-efficacy for schoolwork was measured before and after the course for both face-to-face and distance education formats.

The academic engagement of students was evaluated by a Chinese academic engagement scale before the start of the course and after the final lesson. The Chinese academic engagement scale [15] was revised according to Utrecht Work Engagement Scale-Student developed by Schaufeli [16, 17]. There were 17 items in the Chinese scale, and it was divided into three dimensions: vitality, focus, and dedication. From “completely disagrees” to “completely agrees,” a 7-point scale of 0 to 6 is adopted. The higher the score, the higher the level of academic engagement. The total Cronbach's α coefficient of the scale was 0.951, and the Cronbach's α coefficient of each subscale was 0.858, 0.913, and 0.905.

### Data analysis

SPSS software (version 22.0 for Windows, Chicago, IL) was used for statistical analysis. Continuous variables conforming to a normal distribution were described as mean ± standard deviation, and those that do not conform to the normal distribution were described by quartile. Two independent sample t-tests were used for inter-group comparison. Categorical variables were described by frequency and composition ratios and were compared with the Chi-square test. *P* < 0.05 was considered statistically significant.

## Results

### Comparison of basic demographic data between the two groups of students

Student baseline demographics were similar between the two groups. Except for the average age difference of one year (*p* < 0.001), there was no significant difference between the two groups in sex, home location, only-child status, family economic status, student leadership, last semester’s ranking, and attitude towards the nursing specialty (Table1).

**Table 1:** Student baseline demographics

Variable	Distance learning format group (*n* = 48)	Control group (*n* = 36)	*P* value
Age, mean (SD), yr	19.8(0.7)	20.9(0.4)	**< 0.001**
Sex, Number of boys (%)	10(20.8)	6(16.7)	0.75
Home location			0.094
Countryside	3(6.3)	3(8.3)	
Town	9(18.8)	14(38.9)	
City	36(75)	19(52.8)	
Only child or not			0.106
Yes	36(75)	21(58.3)	
No	12(25)	15(41.7)	
Family economic status			0.462
Good	11(22.9)	5(13.9)	
Medium	35(72.9)	28(77.8)	
poor	2(4.2)	3(8.3)	
Student leaders or not			0.127
Yes	24(50)	24(66.7)	
No	24(50)	12(33.3)	
Last semester's ranking			0.75
Excellent	8(16.7)	4(11.1)	
Good	29(60.4)	24(66.7)	
Poor	11(22.9)	8(22.2)	
Attitude towards Nursing specialty			0.472
Like	10(20.8)	10(27.8)	
Attitude in the middle	34(70.8)	21(58.3)	
Dislike	4(8.3)	5(13.9)	

### Comparison of the students’ academic performance between the two groups

We compared the academic performance of the two groups at the end of the semester and found that the grades of students who received distance teaching during the epidemic period of COVID-19 were significantly lower than those who received face-to-face teaching (Table 2).

**Table 2:** Comparison of the students' grades between the two groups

Variable	Distance learning format group (*n* = 48)	Control group (*n* = 36)	*t*	*p*
Grades	41.48 ± 10.77	50.06 ± 11.90	-3.45	**0.001**

### Comparison of academic self-efficacy between the two groups

At the beginning of the term (baseline), we found that there was no significant difference between the two groups in the total score of academic self-efficacy (*p* = 0.33), the score of learning ability self-efficacy (*p* = 0.47), and the score of learning behavior self-efficacy (*p* = 0.40). Evaluation of academic self-efficacy results at the end of the semester suggests that the score of learning behavior self-efficacy of the students receiving online teaching was significantly lower than that of the students receiving face-to-face teaching (*p* = 0.04). There was no significant difference in the total score of self-efficacy (*p* = 0.72) and the score of learning ability self-efficacy (*p* = 0.84) between the two groups (Table 3).

**Table 3:** Comparison of the academic self -efficacy between the two groups of students

Variable	Distance learning format group baseline (*n* = 48)	Control group baseline (*n* = 36)	*p*	Distance learning format group (*n* = 48)	Control group (*n* = 36)	*p*
Total score of academic self -efficacy	78 (72.25 ~ 85)	71 (66 ~ 88)	0.33	78.5 (72 ~ 85.5)	79.5 (75.25 ~ 85.25)	0.72
Self-efficacy dimension of learning ability	38.5 (34 ~ 42.75)	34 (31.5 ~ 44)	0.47	38.88 ± 6.43	39.17 ± 6.87	0.84
Self-efficacy dimension of learning behavior	40 (37 ~ 43)	37 (33 ~ 44)	0.40	39.15 ± 3.56	41.36 ± 5.40	**0.04**

### Comparison of the academic engagement between the two groups

There was no significant difference in the total score of academic engagement and the scores of each dimension between the two groups at the baseline level (*p* > 0.05). However, the evaluation results at the end of the semester showed that the total score of academic engagement (*p* = 0.04), the score of vitality dimension (*p* = 0.02) and focus dimension (*p* = 0.001) in online teaching students were significantly lower than those of traditional face-to-face teaching students (Table 4).

**Table 4:** Comparison of the academic engagement between the two groups of students

Variable	Distance learning format group baseline (*n* = 48)	Control group baseline (*n* = 36)	*p*	Distance learning format group (*n* = 48)	Control group (*n* = 36)	*p*
Total score of academic engagement	53 (49 ~ 66)	55 (48 ~ 68)	0.83	54.5 (48.25 ~ 66)	62 (53.5 ~ 68.75)	**0.04**
Vitality	18 (17 ~ 22.75)	18 (16 ~ 24)	0.50	19.29 ± 4.912	22.64 ± 7.076	**0.02**
Focus	19 (16.25 ~ 23.75)	18 (16.5 ~ 24)	0.86	19 (17 ~ 22)	23.5(20 ~ 29.5)	**0.001**
Dedication	16.5 (15 ~ 20)	16 (15 ~ 20.5)	0.88	18 (15 ~ 20)	18 (15 ~ 21.5)	0.83

## Discussion

Since the outbreak of COVID-19, online teaching has become an increasingly popular teaching method in medical education all over the world [18, 19]. Teaching converted to a completely online format at our school from March to July in 2020, but no formal evaluation had been performed to compare the performance of students who received traditional face-to-face teaching and distance learning in the Fundamentals of Nursing I course. The present study evaluated the effects of distance education on nursing students’ academic engagement level, learning efficiency level, and academic performance in the nursing fundamentals course. The results of this study showed that the overall course grades were statistically significantly lower in the group of students who received online teaching compared with the students who received face-to-face teaching. In addition, compared with face-to-face teaching, distance teaching greatly reduced their learning behavior self-efficacy and academic engagement. These findings indicate that the pandemic disrupted medical education and training [8] and there are still some problems in the present online teaching mode for the fundamentals of nursing course during the COVID-19 epidemic. We should further find out the causes of these problems and improve teaching in the future to deal with the online teaching mode that may be faced at any time because of the ongoing epidemic situation.

It was surprising that students who studied online through the internet had poor academic performance in the Fundamentals of Nursing I course, especially since the course materials used for both two groups of students were identical and teachers were the same. The main differences between the two groups were the location of the teacher and the background of COVID-19's outbreak in China. Therefore, one hypothesis for the lower course grades on the online teaching group in the spring semester of 2020 could be that this was the first time this course was fully taught through the internet. The faculty could not observe every student, communicate and discuss the course material with students in person, and answer each student's questions effectively [20] as they did in face-to-face teaching. Another hypothesis for lower course grades in the online teaching group could be that COVID-19 was still very serious in China in spring 2020, which brought different degrees of psychological burden and hardships to students and teachers, greatly affecting their concentration on the curriculum [21].

Self-efficacy refers to people's expected judgment on whether they have the ability to complete a certain activity successfully before attempting it [22]. Academic self-efficacy is the application of self-efficacy in the field of learning. It refers to students' expectations and judgment of their learning ability and whether they can successfully complete a learning task in the process of learning activities. According to the theory of Bandura's self-efficacy, students' expectations and judgments about their abilities in the process of learning activities will affect their learning motivation, learning behavior, and academic achievement [23]. When facing new or arduous learning tasks, students with high academic self-efficacy will respond to them actively as challenges while students with low academic self-efficacy will avoid them as difficulties. In our study, the students' learning behavior self-efficacy within an online teaching context was significantly lower than that of face-to-face teaching. On the one hand, this result may be related to the environment of COVID-19 because people under the epidemic are prone to induce psychological reactions such as panic, anxiety, and depression [24, 25], and the excessive tension and anxiety of individuals often lead to the decline of their learning ability in the learning process [26–28]. On the other hand, when learning online, teachers and other students are not around, and they have to solve problems alone, which may also reduce students' learning confidence and learning self-efficacy [29].

Another finding of this study is that the total score of academic engagement, the dimension that measures vitality and focus, was significantly lower for distance-learning students than those of traditional face-to-face teaching students. Academic engagement refers to the willingness to learn, participation, concentration, and subsequent emotions in the learning process; academic engagement can effectively predict students’ current academic performance [30]. A study of middle school students from Turkey found that academic self-efficacy can positively predict students' academic engagement [31]. Another study on online learning showed that students with higher academic self-efficacy are better at using the internet for learning and can actively and effectively participate in online course learning [32]. Therefore, we speculate that academic self-efficacy positively predicts online academic engagement.

The results of this study suggest that during the prevalence of COVID-19, the effect of distance teaching in nursing colleges is not good, and there are great obstacles and challenges in long-term application of distance teaching in the context of normalization of epidemic prevention and control. For example, Kumar A et al.'s research [33] shows that barriers to online learning include low speech and language intelligibility, reliability and connectivity problems, and physical health barriers, such as eye fatigue. Therefore, it is necessary to explore alternative mixed teaching mode and method in distance teaching. Kang HY et al. [34] applied flipped classroom as a mixed education strategy to undergraduate nursing students. The results showed that the mixed teaching model could help students maintain a positive attitude towards learning [35], help learners to learn independently [36], expand the knowledge of participants, and ultimately improve the learning effect of nursing students. Sáiz-Manzanares MC et al. [37] applied the hybrid teaching mode of hypermedia resource learning management system and supervised learning technology in the process of teaching the third year nursing students, and found that it could improve the students' learning outcomes. Based on the above research foundation, it is suggested that in the future, under the general environment of long-term distance education, the application of multiple alternative and mixed teaching models in basic nursing and other professional courses will help to improve students' concentration, learning efficiency and learning engagement, and thus improve the learning effect of nursing students.

In view of the low academic self-efficacy of online learners in this study, it is not difficult to understand the result that academic engagement was also relatively low. Academic self-efficacy, as an important proximal influencing factor of academic engagement, will affect online learners in the face of learning tasks or learning difficulties. These results suggest that future online teaching should invest in student learning by improving their academic self-efficacy and psychological elasticity in the following ways. First, through the intervention of group counseling activities and virtual simulation methods, enhance students' self-esteem, learning interest, and internal motivation. Fundamental Nursing involves the learning and teaching of many basic skills, so the introduction of virtual simulation technology is very important in a resource constrained environment. In recent years, research shows that the application of virtual simulation technology and methods in nursing teaching can achieve good teaching results. Nursing students are satisfied with simulation as a teaching method [38], and think that simulation improves their ability and prepares for professional practice [39]. Green G et al. evaluated the impact of basic nursing practice on nursing students' ability and learning satisfaction at three time points: before simulation (T1), after simulation (T2) and one month after simulation (T3). The results showed that the simulation of basic nursing practice was not only effective immediately after simulation, but also effective one month after simulation [40]. Weiss ME et al. [41] found that using simulation and online learning in the discharge teaching module of nursing students can help students establish patient education skills to improve the prognosis of patients after discharge. To sum up, although there is no report on the application of virtual simulation method in online teaching of fundamental nursing, it is speculated that it will have a positive impact. Second, instructors should pay close attention to their students' learning behaviors in class and give corresponding guidance for learning strategies and methods in order to strengthen students' training by teaching them how to learn more successfully. Third, instructors should pay attention to the emotional changes of their college students, adjust their learning emotions, increase their internal strengths, and better deal with problems in learning. Fourth, programs should try to use synchronous distance education or the combination of synchronous and asynchronous distance education to find the most suitable distance education model for local students.

## Conclusions

Overall, the results of this study suggest that students who received distance learning performed poorly in the Fundamentals of Nursing I course in the context of the COVID-19 pandemic. In the process of online teaching in the future, we should pay more attention to the attempts of synchronous distance teaching and other modes, pay attention to students' emotional experience in learning and the interaction in teaching, and then create a learning atmosphere of joint participation, form effective interventions, and help students experience more positive emotions, so as to improve their academic self-efficacy and academic engagement for better learning and development.

### Limitation

This study has some limitations. It only analyzes the impact of distance education on the academic performance, academic self-efficacy and academic engagement of nursing students in fundamental nursing courses during the prevalence of COVID-19 through a cross-sectional study. Therefore, at present, it is impossible to obtain a clear causal relationship between variables. In the future, we can conduct in-depth research on the mechanism of distance education affecting academic self-efficacy and academic engagement and thus affecting academic performance through longitudinal research. In addition, this research is a quantitative study, which is not conducive to in-depth understanding of the real experience of students when receiving distance education. In the future, it can be further improved by adding qualitative research.

## Supplementary Information

**Additional file 1:** **Supplementary Table1.** Comparison of the topics covered in the discipline of Fundamentals of Nursing 1, in the distance and face-to-face modalities. 

## Data Availability

The dataset analyzed during the current study is available from the corresponding author on reasonable request.
