# Supplementary material for: The impact of distance education on nursing students course performance in a sino-foreign cooperative program during the onset of COVID-19: a quasi-experimental study
Source: BMC Nurs. 2023 Jan 13;22:16. doi: 10.1186/s12912-022-01136-1 (PMC9837464; doi:10.1186/s12912-022-01136-1)
Supplement: Supplementary file 1 — Additional file 1: Supplementary Table1. Comparison of the topics covered in the discipline of Fundamentals of Nursing 1, in the distance and face-to-face modalities. [file 12912_2022_1136_MOESM1_ESM.docx]

Supplementary Table1. Comparison of the topics covered in the discipline of Fundamentals of Nursing 1, in the distance and face-to-face modalities.

| Contents of the Class | Distance Education | Face-To-Face’ Education |
| --- | --- | --- |
| Lesson 1  Infection control-1 | Presentation of the course: objectives, contents, form of evaluation. How to use Network Teaching Platform. How to be a virtual student. Asynchronous recorded lesson on the concept, classification, causes, condition, control, and prevention of nosocomial infection. Discussion on a news about a collective respiratory tract infection case in a children's ward. posted on the platform in the question-and-answer section. | Presentation of the course: objectives, contents, form of evaluation. Lecture on the concept, classification, causes, condition, control, and prevention of nosocomial infection. Discussion on a news about a collective respiratory tract infection case in a children's ward. |
| Lesson 2  Infection control-2 | Asynchronous recorded classroom on different kinds of cleaning, disinfection, and sterilization methods. Critical Analysis Exercise of a nursing treatment measure posted on the platform. | Lecture on different kinds of cleaning, disinfection, and sterilization methods. Critical Analysis Exercise of a nursing treatment measure made, delivered, and presented in class. |
| Lesson 3  Infection control-3 | Virtual visit to the central sterile supply department; Individual led study posted on the platform. The Central Sterile Supply Department Discussion Forum. | Face-to-face visit to the central sterile supply department in a tertiary hospital; Individual directed study and face-to-face discussion about why different sterilization methods are used for different sterile items. |
| Lesson 4  Infection control-4 | Video asynchronous lecture about aseptic and isolation technique.  Literature search on hand hygiene compliance of medical staff to be posted on the platform. Homework: reading Technique standard for isolation in Hospitals in China. | Lecture on aseptic and isolation technique  Discussion on hand hygiene compliance of medical staff in hospitals. Homework: reading Technique standard for isolation in Hospitals in China. |
| Lesson 5  Client Hygiene-1 | Asynchronous recorded classroom on oral care.  Discussion on how to give oral care to a comatose patient in the question-and-answer section of the platform in the question-and-answer section. | Lecture about oral care.  Discussion on how to give oral care to a comatose patient according to nursing process. |
| Lesson 6  Client Hygiene-2 | Reading of book chapter and articles on hair care.  Hand in a video simulating combing hair in bed | Lecture on hair care.  Handing in a video simulating combing hair in bed. |
| Lesson 7  Client Hygiene-3 | Reading text about skin care. Completing the exercises: how to care for a patient with jaundice and itchy skin in liver cirrhosis. | Lecture on skin care. Discussion on a case of how to care for a patient with jaundice and itchy skin in liver cirrhosis. |
| Lesson 8  Client Hygiene-4 | Video asynchronous lecture about the prevention and care of pressure ulcer. Homework about search of the latest pressure ulcer classification system criteria posted on the platform. | Lecture about the prevention and care of pressure ulcer. Homework: search of the latest pressure ulcer classification system criteria. |
| Lesson 9  Client Hygiene-5 | Reading text about perineal care. | Lecture about perineal care. |
| Lesson 10  Client Hygiene-6 | Watching a bed-making video (a closed bed, an anesthetic bed, changing an occupied bed). Discussion about how to prepare a comfortable bed for a patient after surgery posted on the platform in the question-and-answer section. | Lecture on bed making. Discussion on how to prepare a comfortable bed for a patient after surgery. |
| Lesson 11  Sleep and pain management-1 | Video asynchronous lecture about physiological mechanisms, duration, and assessment of sleep, sleep characteristics of hospitalized patients and nursing measures to promote sleep. Homework: five single choice questions about sleep knowledge posted on the platform; reading a literature about sleep. | Lecture on physiological mechanisms, duration, and assessment of sleep, sleep characteristics of hospitalized patients and nursing measures to promote sleep. Homework: five single choice questions about sleep knowledge posted on the platform; reading a literature about sleep. |
| Lesson 12  Sleep and pain management-2 | Reading text about an overview of pain management. Homework: reading a book called Typical Cases of Pain Care. | Lecture on an overview of pain management. Homework: reading a book called Typical Cases of Pain Care. |
| Lesson 13  Sleep and pain management-3 | Asynchronous recorded classroom on factors affecting pain. Discussion on the extent to which emotion contributes to pain posted on the platform in the question-and-answer section. | Lecture about factors affecting pain. Discussion on the extent to which emotion contributes to pain. |
| Lesson 14  Sleep and pain management-4 | Asynchronous recorded classroom on care of pain. Homework about comparing the advantages and disadvantages of different pain assessment tools posted on the platform. | Lecture about care of pain. Homework: to compare the advantages and disadvantages of different pain assessment tools. |
| Lesson 15  Vital signs assessment-1 | Video asynchronous lecture about the assessment and care of body temperature. Completing the homework about how to care for a patient with high fever after surgery posted on the platform. | Lecture on the assessment and care of body temperature. Clinical thinking training: how to care for a patient with high fever after surgery. |
| Lesson 16  Vital signs assessment-2 | Video asynchronous lecture about the assessment and care of pulse. Handing in a video simulating measuring the pulse. | Lecture on the assessment and care of pulse. Handing in a video simulating measuring the pulse. |
| Lesson 17  circulatory and ventilatory support-1 | Video asynchronous lecture about the assessment and care of blood pressure. Discussion on influencing factors of blood pressure measurement posted on the platform in the question-and-answer section. | Lecture on the assessment and care of blood pressure. Discussion on influencing factors of blood pressure measurement. |
| Lesson 18  circulatory and ventilatory support-2 | Reading text about normal breathing and physiological changes. | Lecture about normal breathing and physiological changes. |
| Lesson 19  circulatory and ventilatory support-3 | Video asynchronous lecture about evaluation and care of abnormal breathing. Handing in a video simulating measuring the pulse. | Lecture about evaluation and care of abnormal breathing. Handing in a video simulating measuring the pulse. |
| Lesson 20  circulatory and ventilatory support-4 | Videos about Effective cough, percussion, postural drainage, and aspiration of sputum. Discussion on How to guide a patient to cough effectively after lung surgery posted on the platform in the question-and-answer section. | Lecture about nursing techniques to promote respiratory function (Effective cough, percussion, postural drainage and aspiration of sputum). Communication skill practice: How to guide a patient to cough effectively after lung surgery. |
| Lesson 21  circulatory and ventilatory support-5 | Video asynchronous lecture about oxygen therapy. Homework: 5 short answer questions about oxygen therapy posted on the platform. | Lecture on oxygen therapy. Homework: 5 short answer questions about oxygen therapy. |
| Lesson 22  Nutrition-1 | Video asynchronous lecture about the relationship between diet, nutrition and health, and the relationship between diet, nutrition and disease recovery. | Lecture on the relationship between diet, nutrition and health, and the relationship between diet, nutrition and disease recovery. |
| Lesson 23  Nutrition-2 | Reading text about assessment of nutritional status. Homework about submitting a self-assessment of nutrition posted on the platform. | Lecture about assessment of nutritional status. Homework: submitting a self-assessment of nutrition. |
| Lesson 24  Nutrition-3 | Video asynchronous lecture about hospital diet and special diet care. Discussion on how to make a suitable diet plan for a patient with hepatic encephalopathy posted on the platform in the question-and-answer section. | Lecture about hospital diet and special diet care. Discussion on how to make a suitable diet plan for a patient with hepatic encephalopathy. |
| Lesson 25  Mobility-1 | Video asynchronous lecture about causes of limitation and its impact on the body and assessment of patient activity. | Lecture on causes of limitation and its impact on the body and assessment of patient activity. |
| Lesson 26  Mobility-2 | Asynchronous recorded classroom on assisting patient activities. Discussion on how to develop a bed mobility plan for a patient with a lower extremity fracture posted on the platform in the question-and-answer section in the question-and-answer section. | Lecture on assisting patient activities. Discussion on how to develop a bed mobility plan for a patient with a lower extremity fracture. |
| Lesson 27  Safety-1 | Asynchronous recorded classroom on factors affecting patient safety, assessment of patient safety, common unsafe factors in hospitals, and measures to protect patient safety. Group assignment: the team completed the design of a safe ward and submitted it to the platform. | Lecture about factors affecting patient safety, assessment of patient safety, common unsafe factors in hospitals, and measures to protect patient safety. Group assignment: designing a safe ward. |
| Lesson 28  Safety-2 | Asynchronous recorded classroom on occupational protection of nurses. | Lecture about occupational protection of nurses. |
